# The Regulating Role of Nano-SiO_2_ Potential in the Thermophysical Properties of NaNO_3_-KNO_3_

**DOI:** 10.3390/nano15241854

**Published:** 2025-12-11

**Authors:** Manting Gu, Dan Zhang, Chuang Zhu, Panfeng Li, Wenxin Han

**Affiliations:** 1 School of Energy and Electrical Engineering, Qinghai University, Xining 810016, China; 2 College of Engineering, Qinghai Institute of Technology, Xining 810016, China

**Keywords:** molten salt, specific heat, mechanism, nanoparticles, thermophysical properties, density functional theory

## Abstract

Molten salt, as a phase change heat storage material, can be used to mitigate the volatility of clean energy. Increasing the specific heat of molten salts can help to increase heat storage density and reduce costs. In this study, nanoparticles with different potentials were prepared and doped into Solar Salt (NaNO_3_-KNO_3_). The modification results of the nanoparticles were evaluated by transmission electron microscopy, energy dispersive X-ray spectroscopy and infrared spectroscopy, and the modification process was analyzed by density functional theory. The specific heat, thermal diffusion coefficient, melting point, latent heat of the composites and their variation mechanism were analyzed using synchronized thermal analyzer, laser flash analyzer and scanning electron microscope. It was found that acidification was able to modify the SiO_2_ nanoparticles and that the higher the acidity, the more the negative charge of the nanoparticles was neutralised. A 25.8% decrease in zeta potential to −23.17 mV was observed for the nano-SiO_2_ after treatment with HCl at pH 1, compared to the non-acidified sample. The microelectric field generated by the charged nanoparticles affects the thermophysical properties such as the specific heat of the molten salt-nanoparticle composites, with one of the samples having the largest specific heat (1.79 J/(g·K)) and thermal diffusion coefficient (0.94 mm^2^/s), which were increased by 13.3% and 14.6%, respectively, compared to the Solar Salt. This study attributes the alterations in thermophysical properties to the variation in ion separation distance induced by the charge on nanoparticles.

## 1. Introduction

Solar energy has emerged as a prominent focus of research and development as a form of clean, renewable energy [1]. However, its instability and intermittent nature during day and night limit its ability to provide continuous and stable power supply. In the context of global energy structure transformation and the rapid development of renewable energy, thermal storage technology is regarded as an important means to smooth out the fluctuation of energy supply and demand, and to enhance the efficiency of energy utilization [2]. Enhancing the performance of thermal energy storage (TES) materials is pivotal to advancing TES technologies. As a phase-change TES material, molten salt offers significant advantages, including relatively low cost, a broad operating temperature range, and excellent thermal stability. These properties establish it as a highly promising solution for large-scale TES applications, effectively balancing economic viability with practical utility [3].

Among the many molten salt systems, nitrate-based molten salts have been used on a large scale in fields such as concentrated solar power due to their excellent thermophysical properties [4]. For example, a thermal storage material, Solar Salt, consisting of 60% NaNO_3_ and 40% KNO_3_ (mass fraction) has been used as a commercial heat storage and heat transfer medium since 1937 [5]. Although molten salt shows good application prospects in heat storage, there is still room for optimisation in terms of heat storage density and heat transfer performance. In recent years, researchers have found that doping molten salts with nanoparticles often improves their heat storage or heat transfer properties. Navarrete et al. [6] evaluated four methods for preparing solar salt-based nanofluids containing 1 wt% SiO_2_ nanoparticles and reported that all four methods succeeded in enhancing the specific heat capacity, with a maximum increase of 21.1%. Nithiyanantham et al. [7] investigated the influence of spherical and rod-shaped nano-Al_2_O_3_ on the thermophysical properties of the 51 wt% NaNO_3_-49 wt% KNO_3_ mixture. They found that the composite doped with spherical nano-Al_2_O_3_ exhibited 7% and 6% enhancement in the specific heat capacity in the solid and liquid states, respectively, compared to the base salt. Aljaerani et al. [8] investigated a ternary nitrate salt doped with CuO nanoparticles at concentrations of 0.1, 1, 3, and 5 wt%. Their tests determined that the 0.1 wt% concentration was the optimal dosage, resulting in an enhancement of the specific heat capacity by 5.6% and the latent heat by 30%. In a subsequent study, Aljaerani et al. [9] doped a ternary nitrate salt with various concentrations of nano-TiO_2_. They reported that a 0.1 wt% addition could enhance the specific heat capacity of the sample by 5.5%. Yu et al. [10] reported that the co-doping of nano-SiO_2_ and nano-TiO_2_ into a quaternary nitrate mixture resulted in a 28.1% enhancement in specific heat capacity relative to the base salt.

Although research on the enhancement of molten salt properties by nanoparticles is extensive, a definitive consensus regarding the underlying mechanism has not been reached. Inspired by the existing mechanisms of semi-solid layers [11,12], interfacial thermal resistance [11,13,14] etc., we believe that the charged state of nanoparticles may be another new and important factor for specific heat enhancement. However, current research on the effect of nanoparticle surface charge on the thermophysical properties of molten salts primarily focuses on simulation studies [15,16], while experimental validation remains relatively scarce. In this study, Solar Salt and SiO_2_ were selected as the research materials primarily due to their low cost and extensive research background. Current research indicates that a nanoparticle doping concentration of 1 wt% typically yields the most significant enhancement in the specific heat capacity of molten salts [17,18,19]. This optimal doping ratio is similarly observed in studies focusing on nano-SiO_2_ incorporated in solar salt systems. Accordingly, this study adopted 1 wt% as the standard doping concentration for sample preparation [20,21]. This established foundation facilitates straightforward comparison and analysis of our findings with existing literature. In this study, the nanoparticles were characterised by transmission electron microscopy, X-ray photoelectron spectroscopy and zeta potential analyzer, and the process of nanoparticle modification was investigated by density functional theory (DFT). The heat storage and heat transfer properties of the molten salt nanocomposites were tested using a simultaneous thermal analyzer and a laser flash analyzer. Finally, the nano-molten salt composites were characterised microscopically by scanning electron microscopy. This study experimentally investigates the influence of nanoparticles with different electrical potentials on the thermophysical properties of molten salts, as opposed to relying solely on simulations. The analysis from the perspective of micro-electric fields generated by the nanoparticles provides new insights into the mechanisms underlying the specific heat capacity changes. This approach offers a fresh perspective for future in-depth mechanistic studies on specific heat enhancement of molten salts in the field.

## 2. Experiment

### 2.1. Sample Preparation

#### 2.1.1. Modification of Nano-SiO_2_

**Figure 1:**
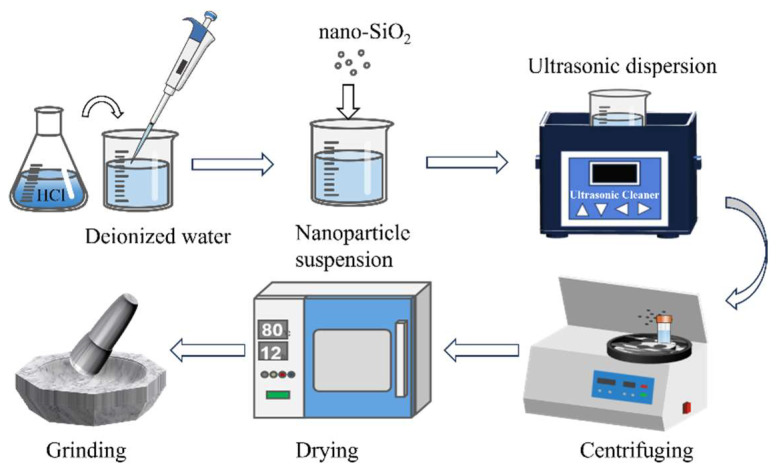
Preparation process of modified SiO_2_.

The materials used in the preparation process included: dilute hydrochloric acid, deionised water, nano-SiO_2_ (99.9%, Aladdin Biochemical Technology Co., Ltd., Shanghai, China) with a thermal conductivity of 8.3 W/(m·K) and a specific heat of 0.99 J/(g·K). Figure 1 shows the preparation process of modified SiO_2_. First, an acidic solution with a pH of 1 was prepared by titrating 120 mL of deionized water with dilute hydrochloric acid. Second, 0.5 g of unmodified nano-SiO_2_ (named NP) was dispersed in the solution, ultrasonicated for 5 min at room temperature and then transferred to a centrifuge tube. It was then centrifuged for 5 min at 7000 r/min, after which the supernatant was decanted. The precipitate was dried in an oven at 80 °C for 12 h, then ground and reserved. SiO_2_ nanoparticles were subjected to modification under acidic conditions at pH 3 and pH 5, respectively, following the aforementioned procedure. The three types of nanoparticles prepared above were labeled NP-1, NP-3, and NP-5.

The modification process of the nanoparticles was analyzed using Gaussian16 (Version G16 A.03) [22]. This program simulates the energy, structure, and various properties of molecular systems by solving the Schrödinger equation, enabling the following tasks: molecular structure and energy calculations, prediction of molecular properties, and simulation of chemical reactions. In this study, a structural model of SiO_2_ was constructed, and the reaction process between HCl and SiO_2_ was analyzed based on the calculated energy barriers and transition state structures. The computational procedure was based on density functional theory (DFT) using the B3LYP functional with the 6-311g(d,p) basis set [23,24,25,26]. Transition states were identified by the presence of a single imaginary frequency. Geometry optimization for reactants and products, as well as transition state searches, were performed using the Berny optimization algorithm. All calculations were carried out at 298.15 K and 101.325 kPa. Reaction energy barriers are expressed in terms of Gibbs free energy changes.

#### 2.1.2. Preparation of Composite Materials

**Figure 2:**
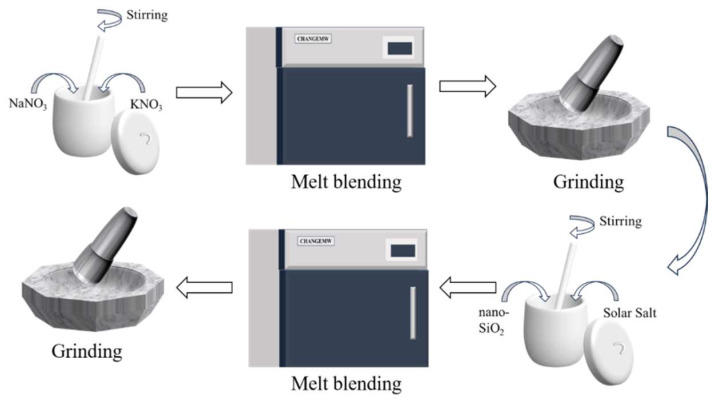
Preparation process of molten salt-nanoparticle composites.

The materials used in the preparation process included NaNO_3_, KNO_3_, and modified nanoparticles. The two nitrate salts were purchased from Aladdin Biochemical Technology Co., Ltd., and their basic properties are listed in Table 1 [27]. To synthesize Solar Salt (labeled SS), a 6:4 mass ratio blend of NaNO_3_ and KNO_3_ was blended and subsequently heated in a microwave muffle furnace at 250 °C for 30 min. After cooling, the resulting product was ground into a powdered form for use. In the next step, 0.05 g of nano-SiO_2_ and 4.95 g of SS were weighed, mixed well and put into a muffle furnace, held at 250 °C for 30 min, and then taken out and milled after cooling to obtain the molten salt nanocomposite materials (labeled MN). This preparation process is shown in Figure 2. The samples obtained by compounding NP-1, NP-3, NP-5, and NP with SS were labeled as MN-1, MN-3, MN-5, and MN, respectively.

### 2.2. Measurement

#### 2.2.1. Zeta Potential

Zeta potential tests were performed using a Malvern Zetasizer Nano (DynaPro NanoStar, Wyatt Technology, Goleta, CA, USA) for NP-1, NP-3, NP-5, and NP, respectively. An appropriate amount of powder sample was dispersed in deionised water to prepare a suspension with a mass fraction of 0.1%, and ultrasonicated for 3 min for uniform dispersion. In dynamic light scattering measurements, the scattering angle was set at 90° and the temperature at 25 °C. The accuracy of the test results was ensured by observing the electrophoretic mobility distribution profile as well as the zeta potential distribution profile during the test. The measurements were repeated three times for each group of samples.

#### 2.2.2. Morphology and Elemental Content of Nanoparticles

Nanoparticles were analyzed in microregions using transmission electron microscopy (TEM, FEI-Talos F200S, Thermo Fisher, Waltham, MA, USA) combined with energy dispersive X-ray spectroscopy (EDS). Nanoparticle dispersions were prepared using water as the dispersant, and the solution was dropped onto a copper mesh and dried. The acceleration voltage was 200 kV. The functional groups of the nanoparticles were analyzed using a Fourier transform infrared spectrometer (FTIR, Nicolet 6700, Thermo Fisher, Waltham, MA, USA). The powder sample was first subjected to a pelletizing process. After collecting a background scan, the sample was placed in the instrument. For each sample, the mass ratio of nanoparticles to KBr was maintained at 1:100. The pellets were prepared under a pressure of 15 MPa, which was applied for 5 min. The measurement method was the transmission method with the resolution set to 4 cm^−1^, the number of sample scans was 32, and the scanning range was 4000–400 cm^−1^.

#### 2.2.3. Thermal Storage Properties of MNs

The specific heat measurements were conducted using a synchronous thermal analyzer (STA-449F3, NETZSCH, Selb, BAV, Germany) with aluminum crucibles. The first step is baseline measurement. A crucible was placed in the sample and reference holders of the STA instrument, respectively, and the temperature control program was set to execute the following sequence: (1) maintained isothermally at 260 °C for 15 min; (2) heated to 300 °C at a rate of 10 °C/min; (3) held isothermally for 15 min. Nitrogen served as both the protective gas (20 mL/min) and the purge gas (50 mL/min). The second step is the sapphire reference measurement. A sapphire standard sample (diameter: 4 mm; mass: 12.58 mg) was placed in the sample aluminum crucible. Its DSC curve was measured using the baseline obtained in the previous step. The third step is sample measurement. The sapphire was removed and a measured quantity of the sample was then loaded into the same crucible for DSC curve acquisition. In this step, the masses of MN-1, MN-3, MN-5, and MN are 7.64 mg, 7.46 mg, 7.69 mg, and 7.31 mg, respectively. Finally, utilizing NETZSCH’s proprietary analysis software (Version 8.0.2), the specific heat capacity of the samples was determined. All measurements were conducted in triplicate for each sample.

The DSC curves of the phase transition process of the samples were also done by STA-449F3. The test temperature range was 160–280 °C, the heating rate was 10 °C/min. The flow rates of nitrogen as purge gas and protective gas were 50 mL/min and 20 mL/min, respectively. All of the above testing processes were performed three times to ensure the accuracy of the data.

#### 2.2.4. Heat Transfer Properties of MNs

The thermal diffusion coefficients of the samples were measured using a laser flash analyzer (LFA-467HT, NETZSCH, Selb, BAV, Germany). Approximately 0.20–0.25 g of the sample was placed in a 12.7 mm diameter platinum-rhodium crucible and heated on a heating plate until the sample was completely molten. Cover the crucible so that the bottom of the crucible lid is in contact with the molten sample and does not overflow. The crucible was transferred to a room temperature environment and left to solidify for 5 min. After solidification, the graphite coating was uniformly sprayed on the top and bottom surfaces of the crucible and then tested. The purge and protective gases for the measurement process are high-purity argon with a flow rate of 50 mL/min. The test temperature was 300 °C. Three replicate measurements were made for each sample.

#### 2.2.5. Micro-Morphological Characterisation of MNs

A field emission scanning electron microscope (JSM-7900F, JEOL, Akishima, Japan) was used to observe the microscopic morphology of the samples. Prior to testing, the samples powder was fixed onto a conductive adhesive, and then they were sprayed with gold using an ion sputter coater (KYKY SBC-12, Zhongke Technology Co., Ltd., Beijing, China). The accelerating voltage during the test was 3.0 kV.

## 3. Results and Discussion

### 3.1. Modification of SiO_2_ Nanoparticles

**Figure 3:**
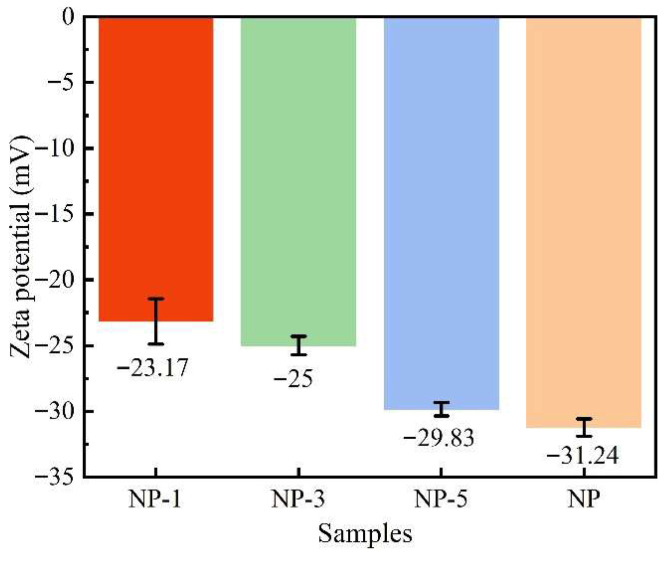
Zeta potential of SiO_2_ samples.

The zeta potentials of different nanoparticle samples are shown in Figure 3. As the acidity of the HCl solution increases, the zeta potential of the silica nanoparticles becomes less negative, changing from −31.24 mV for the untreated sample to −29.83 mV at pH 5 and −23.17 mV at pH 1. This reduction in the magnitude of the negative zeta potential weakens the electrostatic repulsion between particles, thereby increasing their tendency to agglomerate. The decrease in the negative zeta potential can be attributed to the presence of silanol groups (Si-OH) on the nanoparticle surface. In more acidic environments, a greater number of Si-OH groups become protonated, forming Si-OH_2_^+^ species [28].

**Figure 4:**
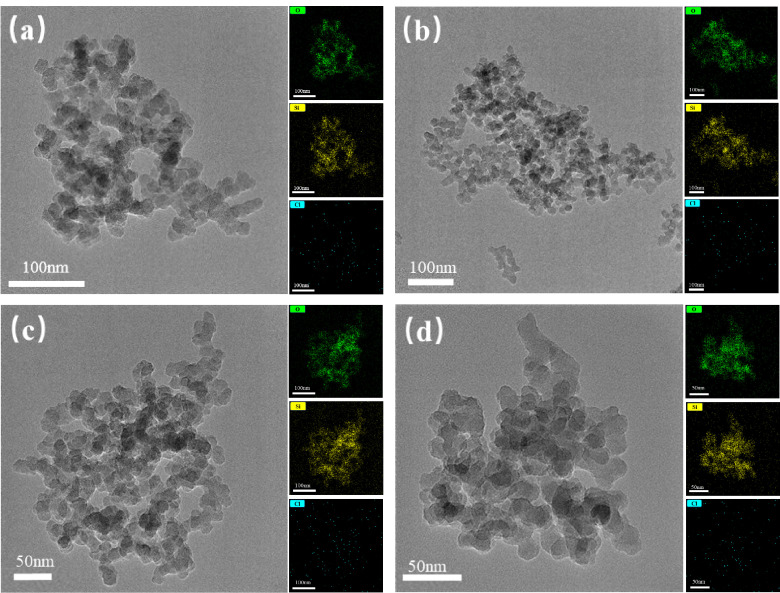
Micro-morphology and elemental distribution of (**a**) NP-1, (**b**) NP-3, (**c**) NP-5 and (**d**) NP.

The morphology and elemental content of the nanoparticles were analyzed by TEM-EDS (see Figure 4 and Table 2). The SiO_2_ nanoparticles were spherical with a uniform particle size (~20 nm). In terms of elemental content, although the atomic contents of NP-5 and NP-3 are close to each other (which may be related to the 0.1% error in the equipment itself), the overall trend is still reflected in the fact that the higher the acidity of the modification process, the smaller the O content and the larger the Si content of the resulting nanoparticles will be. For example, NP has the largest amount of O atoms (71.58%), while NP-1 has the smallest (68.9%). Because O on the surface of the nanoparticle is negatively charged and Si is positively charged, the overall negative potential presented by the nanoparticles decreases when the Si content increases and the O content decreases, which is consistent with the rule of the potential in Figure 3.

**Figure 5:**
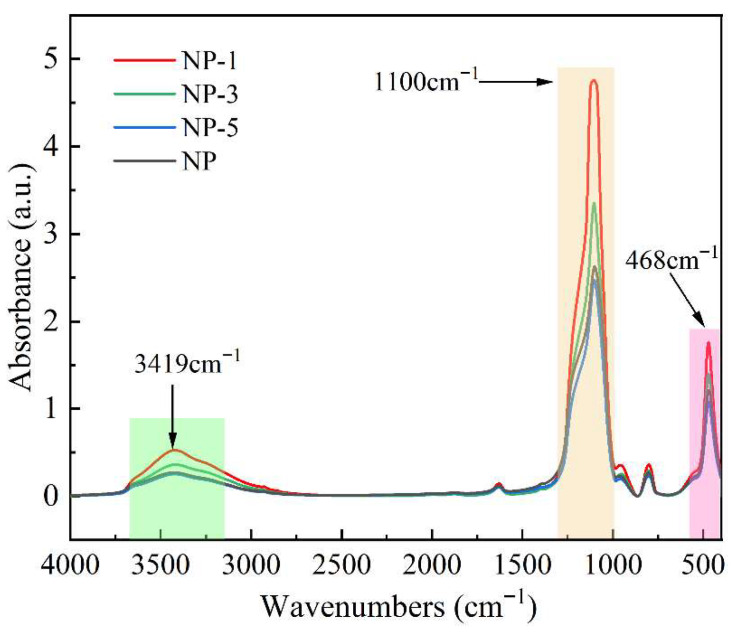
Infrared spectrum of SiO_2_.

The chemical bonds of the nanoparticle samples were further analyzed by FTIR. Figure 5 shows the absorbance of different nanoparticles during the wavelength change in continuous infrared light. The peak at 3419 cm^−1^ corresponds to the stretching vibration of O-H at different positions on the protonated SiO_2_ surface [29], and the two peaks at 1100 cm^−1^ and 468 cm^−1^ correspond to the telescopic and bending vibrations of O-Si-O [30], respectively. As can be observed, the acidity of the solution used for modification positively correlates with the abundance of O-H and Si-O-Si bonds in the resulting nanoparticles. The increase in O-H bonds can be attributed to the protonation process, which inherently increases the number of such bonds. The increase in Si-O-Si bonds can be attributed to the protonation of surface Si-OH groups to form Si-OH_2_^+^, which leads to an expansion of Si-O bond distances. This structural change enhances the participation of surface silicon atoms in the vibrational modes of Si-O-Si networks located within the nanoparticle’s subsurface layer. The observations from the FTIR spectra are consistent with the previously discussed zeta potential trends.

**Figure 6:**
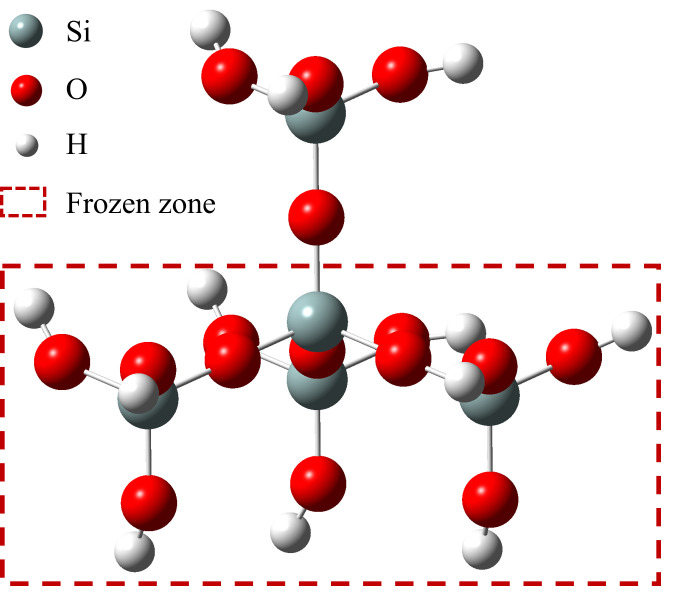
Crystal structure of SiO_2_.

The SiO_2_ structural model in Figure 6 was constructed using crystallographic data from the Crystallography Open Database [31]. To ensure bonding integrity, hydrogen atoms were added to the structure. Since it is the atoms on the surface that are primarily involved in the reaction, the other secondary atoms are frozen during the calculation (atoms in the red border in Figure 6). Based on DFT, geometrical structures of the three molecular configurations involved in the reaction between SiO_2_ and HCl were optimized at the B3LYP/6-311G (d, p) level of theory. Additionally, frequency calculations were performed on the transition state structures.

**Figure 7:**
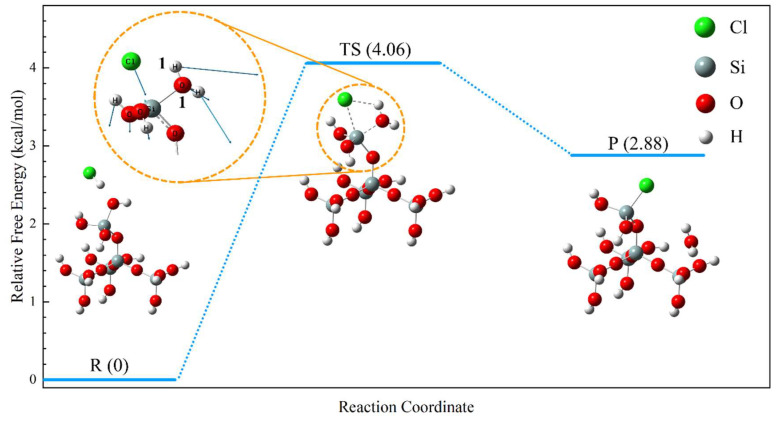
DFT-calculated reaction pathway for nanoparticle modification.

The relationship between reaction paths and molecular energies is shown in Figure 7. The reaction pathway, as shown in Figure 7, includes the reactant (R), the transition state (TS), and the product (P). The reaction process involves H^+^ in solution combining with -OH on Si-OH to form the H_2_O and Cl^-^ combining with Si to form the Si-Cl chemical bond [32,33,34]. There is one transition state in this process, which contains only one imaginary frequency (−112.96 cm^−1^), and the vibrational orientation of the atoms in the TS gives the configuration a clear convergence to the structure of the product. Information on the spacing of key atoms at the reaction site for R, TS and P is shown in Table 3. The relative Gibbs free energy of P is 2.88 kcal/mol higher than that of R, indicating that this reaction is thermodynamically unfavorable. This interpretation is consistent with the absence of substantial Cl signals in both EDS and IR analyses.

### 3.2. Specific Heat

**Figure 8:**
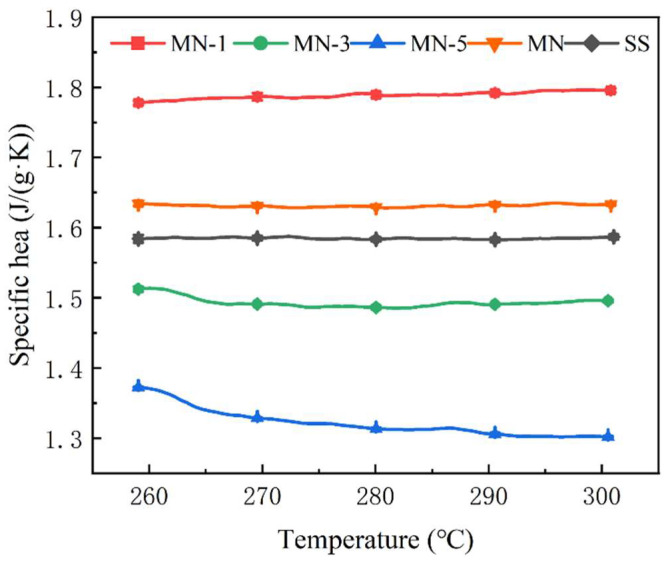
Specific heat of solar salt and composites.

Figure 8 shows the specific heat curves of these five samples in the molten state. The error bars in the graph represent the standard error. The average specific heat of SS is 1.58 J/(g·K) and that of MN is 1.63 J/(g·K), which is in agreement with the results of the current study [35,36]. The average specific heats of MN-1, MN-3, and MN-5 are 1.79 J/(g·K), 1.49 J/(g·K), and 1.32 J/(g·K). It is clear that the nanoparticles treated with a solution of pH 1 have a significant strengthening effect on the specific heat of SS, which can be increased by 13.3%, and also by 9.8% more than that of the material obtained in the traditional way (doping with unmodified nanoparticles). However, it is also noted that instead of increasing the specific heat of SS, the specific heat of MN-3 and MN-5 decreases it. Obviously, with the same nanoparticle type and doping amount, this variation is only related to the modification conditions of the nanoparticles.

**Figure 9:**
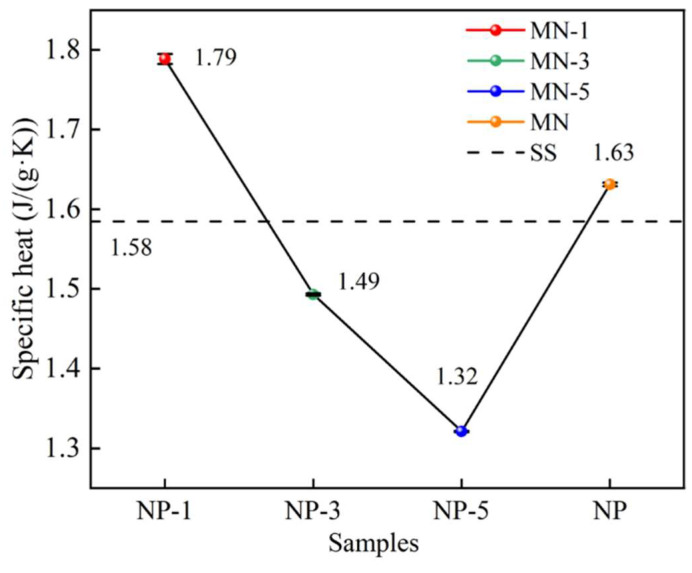
Average specific heat of the samples versus SiO_2_ at different potentials.

To probe the origin of this phenomenon, the average specific heat of the sample was correlated with the nanoparticle charge (see Figure 9). A pattern can be seen, in the order of MN-1, MN-3, and MN-5, as the nanoparticle potential increases, the specific heat of the composites shows a significant decrease, while the specific heat of MN shows a rebound. Given the lack of consensus on a deep microscopic mechanism for this phenomenon, this work undertakes a novel discussion, guided by the established frameworks within the field.

It should be noted that the following discussion is based on a hypothesis derived from the well-established Dulong-Petit law, which states that at room temperature, the molar heat capacity of most solid elements is approximately constant, with a value of about 25 J/(mol·K). This is because atoms in the solid state are bound to fixed positions and can only vibrate, and the heat capacity can be estimated in terms of the number of degrees of freedom according to the energy equalization theorem. However, for substances in the molten state, because of the presence of large translational kinetic energies of particles, structural rearrangements, significant anharmonic vibration, etc., not all of the heat absorbed by the material is used to increase the kinetic energy of the molecules or particles, but is stored in the potential energy. Therefore, the specific heat of a substance in the molten state is generally larger than that of a solid [37,38,39], and it is not possible to quantitatively estimate the specific heat of a substance in the molten state in terms of 3R (R is the ideal gas constant). But one thing that can be considered qualitatively for both solid and molten materials is that when the specific heat is compared using the unit of mass heat capacity rather than molar heat capacity, the greater the molar mass of the material, then the smaller the value of its mass heat capacity will be. In other words, for a material per unit mass, if it contains a greater number of particles (the greater the number of moles), then it tends to have a greater specific heat of mass. Therefore, it is conjectured that the addition of a small amount of nanoparticles introduces alterations to the microstructure of the molten salt, causing it to exhibit an effect similar to that of a material containing more particles per unit mass. Thus, during heating and cooling, a portion of the heat is absorbed or released in the form of potential energy, a process that leads to an increase in specific heat.

As for MN-1, which is the composite of NP-1 and SS, the negatively charged nanoparticles electrostatically attract K^+^ and Na^+^ ions while repelling NO_3_^−^ ions. This interaction disrupts the original vibrational equilibrium of the cations and anions within the molten salt, consequently increasing the separation between ions of opposite charge. Consequently, the vibration and motion of the ions become less influenced by the surrounding counter-ions. In other words, while cations and anions originally moved as a coupled system, they now exhibit a tendency toward independent motion. This microstructural change corresponds to the aforementioned phenomenon where “a greater number of particles are contained per unit mass of the material.” The change in the distance between the anions and cations inevitably leads to a change in the potential energy, which is generated precisely due to the microelectric field generated by the charge carried by the nanoparticles. Thus, the specific heat of MN-1 is substantially enhanced.

For MN-3 and MN-5, the nanoparticles included in the material have a greater potential but result in a lower specific heat of the composite. The underlying reason is hypothesized as follows: while an increased potential further separates cations from anions, it simultaneously reduces the distance between ions in the Stern layer and the nanoparticle surface. Due to spatial confinement, this reduction compresses the interionic distance among similarly charged ions within the layer. As a result, the motion of ions becomes more strongly influenced by their neighbors, thereby weakening their tendency toward independent motion. Consequently, a higher potential leads to a more pronounced reduction in specific heat capacity.

For MN, it shows an increase in specific heat with further increase in potential. The reason for this is surmised to be as follows: the effect of highly charged nanoparticles on their surface Stern layer is the same as that of MN-3 and MN-5, and likewise leads to a decrease in the specific heat. On the other hand, a higher potential also induces a stronger repulsive effect within the Shear layer, forcing NO_3_^−^ ions to move farther away from the nanoparticle surface. This increased distance allows these displaced anions to act as new negative charge centers, which subsequently attract surrounding metal cations while repelling other anions. Although these displaced NO_3_^−^ ions are small in size, their population becomes substantial due to the high potential of the nanoparticles. Consequently, a significant number of counter-ions are influenced by these NO_3_^−^ ions. The increased separation among these affected counter-ions enhances their tendency toward independent motion, thereby leading to the observed increase in the specific heat of the MN composite.

### 3.3. Thermal Diffusion Coefficient

Table 4 presents the thermal diffusivities of the five samples in the molten state. Evidently, the composites containing nanoparticles all exhibit higher thermal diffusivities than the SS. Notably, MN-1 shows the highest value, being 1.15 times that of SS. This enhancement can be attributed to the inherently high thermal diffusivity of the solid nanoparticles themselves, which positively contributes to the overall thermal transport capability of the composite.

However, within the composite series, the thermal diffusivity is observed to decrease as the potential of the nanoparticles increases. The underlying mechanism is that nanoparticles with a higher potential attract metal ions more tightly and influence a greater number of them, leading to the formation of larger ionic aggregates. The speed of heat transfer through these larger aggregates is presumably slower than that through smaller molecular entities.

### 3.4. Melting Point and Latent Heat

**Figure 10:**
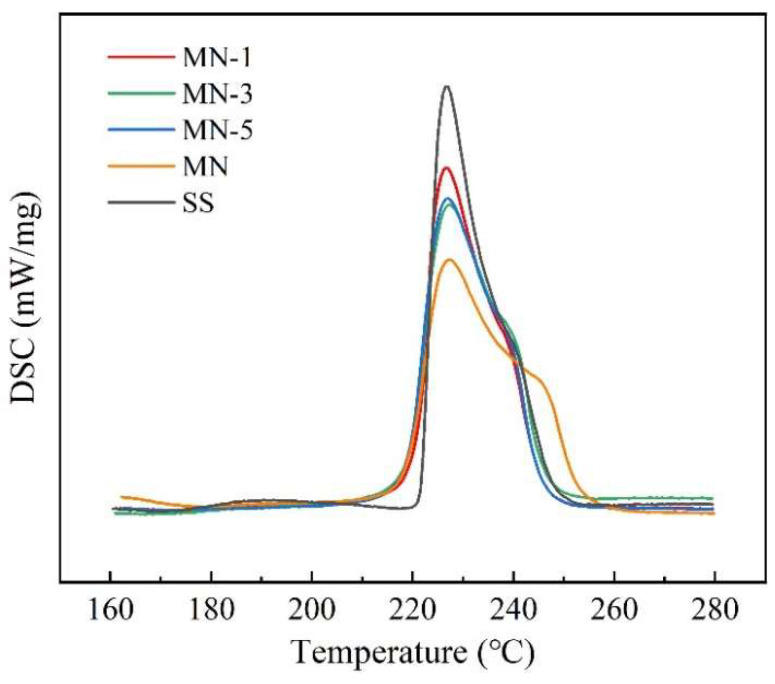
DSC curves of the samples.

The melting points and latent heats of phase change for the samples, obtained from the DSC curves in Figure 10, are listed in Table 5. For the SS, the measured melting point and latent heat are 221.7 °C and 114.8 J/g, respectively, closely matching the values found in the literature [21].

In terms of melting point, the incorporation of nanoparticles leads to larger spacing of positive and negative ions in the molten salt and smaller bonding energy, so the melting points of the composites are all lower than that of the base salt. The effect of nanoparticles on ion spacing is related to their potential; the higher the potential, the lower the melting point, due to the fact that nanoparticles with large potentials can reduce the lattice energy to a greater extent.

Regarding the latent heat, the incorporation of nanoparticles generally leads to a reduction in the latent heat of the molten salt. This decrease can be attributed to two primary reasons. First, the nanoparticles themselves occupy a portion of the mass that would otherwise be salt, effectively diluting the phase change material. Second, the charged surfaces of the nanoparticles influence the ionic environment. During the solidification of the molten salt, the cations and anions near the nanoparticle surfaces maintain certain structural features reminiscent of the molten state, such as an electric double layer distribution, or even become bound to the nanoparticles. This configuration reduces the number of chemical bonds formed between the positive and negative ions in the solid matrix. Consequently, when the solid salt is heated and melts, the total amount of heat required for this phase transition is diminished, resulting in a lower measured latent heat.

For the composite materials, a trend is observed where a higher nanoparticle potential corresponds to a lower latent heat. This is because nanoparticles with a greater potential cause a more significant reduction or weakening of chemical bonds within the system. Concurrently, the stronger electric field associated with a high potential more substantially disrupts the uniformity of ion distribution around the nanoparticles. This disruption leads to the separation of the phase transition peak, which is consistent with the observed broadening of the melting peak in the DSC curve of MN in Figure 8.

**Figure 11:**
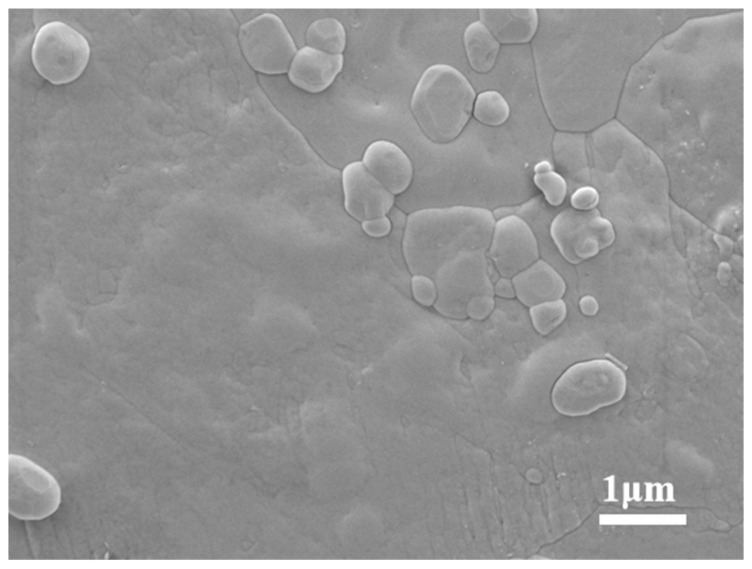
Microscopic morphology of solar salt.

**Figure 12:**
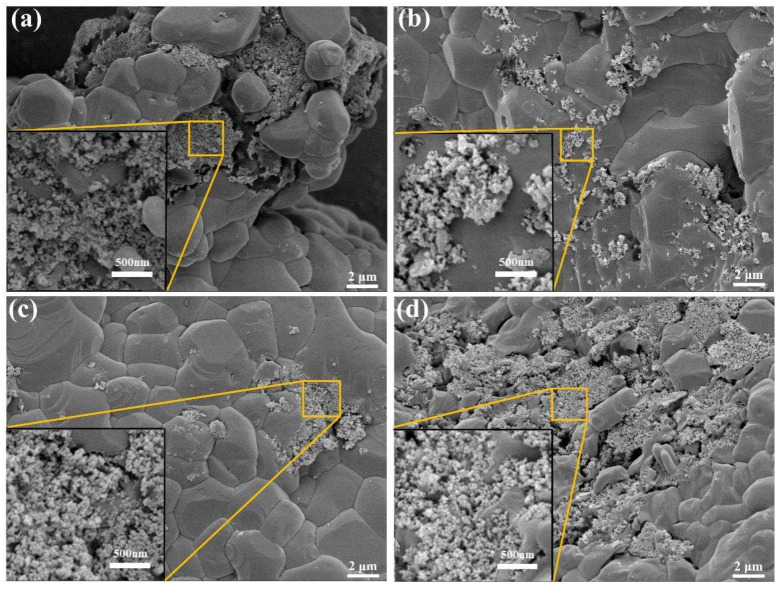
Microscopic morphology of samples of solar salts composited with different SiO_2_: (**a**) MN-1, (**b**) MN-3, (**c**) MN-5 and (**d**) MN.

In addition, the reduction in melting point and latent heat of phase transition of the composites was also analyzed from the micro-morphological point of view. Figure 11 shows the microscopic morphology of the solar salt, which can be seen to have a very flat surface with no obvious special structure. Figure 12 shows the surface morphology of the molten salt containing different nanoparticles. It can be seen that in the nanoparticle-enriched regions, the molten salts are intermingled with the nanoparticles, creating holes in the smooth surface. The molten salt within these regions, being in direct contact with the nanoparticle surfaces, undergoes melting more readily during the heating process.

## 4. Conclusions

The influence of the microelectric field generated by SiO_2_ nanoparticles on the thermophysical properties of Solar Salt was systematically investigated through the modulation of their surface charge. A potential novel mechanism, by which the specific heat capacity of molten salts is affected by nanoparticles, was specifically discussed. The main findings of this study are summarized as follows:

(1) The surface charge of nano-SiO_2_ can be modified through acidification, and a higher acidity leads to a greater neutralization of their negative zeta potential. The potential of the unacidified nanoparticles in deionized water was −31.24 mV, whereas after treatment with hydrochloric acid at pH 1, the potential was reduced to −23.17 mV. The acidification process was found to induce changes in the surface elemental content and chemical bonds of the nanoparticles.
(2) The specific heat of molten salt-nanoparticle composites can be affected by the microelectric field generated by charged nanoparticles. A specific heat capacity of 1.79 J/(g·K) was achieved for the molten salt when nanoparticles treated with HCl at pH = 1 were incorporated, representing a 13.3% enhancement compared to Solar Salt. However, it was also observed that the specific heat of Solar Salt was reduced to varying degrees by nanoparticles treated at pH = 3 and pH = 5. In this study, it is suggested that the effect of the nanoparticle charge on the spacing of the surrounding positive and negative ions may be an important factor contributing to this phenomenon of specific heat change.
(3) The thermal diffusivity, melting point, and latent heat of the composite material are also influenced by the effect of nanoparticle surface charge on the separation between surrounding cations and anions. The underlying mechanisms are associated with the extent of ion aggregation, as well as the bonding energy and quantity of chemical bonds between cations and anions.

## Figures and Tables

**Table 1:** Basic parameters of raw materials [27].

Raw Material	Melting Point (°C)	Density (g/cm^3^)	Latent Heat (J/g)	Purity (%)
NaNO_3_	310	2.26	173	≥99.0
KNO_3_	337	2.11	115	≥99.0

**Table 2:** Atomic fraction of SiO_2_ samples (%).

Element Types	NP-1	NP-3	NP-5	NP
O	68.90	70.91	70.83	71.58
Si	31.00	28.78	28.94	28.30
Cl	0.10	0.31	0.24	0.11

**Table 3:** The critical interatomic distance at the reaction site (Å).

Molecule	Si-O (1#)	O (1#)-H (1#)	H (1#)-Cl	Cl-Si
R	1.66	1.77	1.32	4.08
TS	1.86	0.99	2.12	2.51
P	6.04	0.96	5.53	2.06

1# represents atoms specified in Figure 7.

**Table 4:** Thermal diffusion coefficients of samples at 300 °C.

Samples	MN-1	MN-3	MN-5	MN	SS
Thermal diffusivity (mm^2^/s)	0.940 (±0.003) ^a^	0.917 (±0.002) ^a^	0.875 (±0.000) ^a^	0.845 (±0.000) ^a^	0.821 (±0.002) ^a^

^a^ is the standard error.

**Table 5:** Melting point and latent heat of the samples.

Samples	MN-1	MN-3	MN-5	MN	SS
Melting point (°C)	220.6 (±0.2) ^a^	219.1 (±0.1) ^a^	219.3 (±0.1) ^a^	218.9 (±0.1) ^a^	221.9 (±0.1) ^a^
Latent heat (J/g)	106.0 (±0.2) ^a^	105.8 (±0.1) ^a^	105.1 (±0.1) ^a^	102.8 (±0.2) ^a^	113.7 (±0.3) ^a^

^a^ is the standard error.

## Data Availability

The data presented in this study are available on request from the corresponding author. The data are not publicly available due to lack of authorization from the funding institution.
